# Ten-year experience on home care for patients with plasma cell disorders: bringing optimal therapy home

**DOI:** 10.1007/s10238-025-01683-2

**Published:** 2025-05-27

**Authors:** Flavia Bigi, Mauro Fiacchini, Roberta Restuccia, Simone Masci, Gaia Mazzocchetti, Marco Talarico, Michele Puppi, Ilaria Sacchetti, Enrica Manzato, Miriam Iezza, Katia Mancuso, Chiara Sartor, Lucia Pantani, Paola Tacchetti, Rania Abd Alatif, Erica Pastore, Simona Barbato, Michele Cavo, Elena Zamagni, Ilaria Rizzello

**Affiliations:** 1 IRCCS Azienda Ospedaliero-Universitaria Di Bologna, Istituto Di Ematologia “Seràgnoli”, Bologna, Italy; 2 Dipartimento Di Scienze Mediche E Chirurgiche, Università Di Bologna, Bologna, Italy; 3 Associazione Italiana Contro Le Leucemie, Linfomi e Mieloma Di Bologna, Via Massarenti 9, 40138 Bologna, BO Italy

**Keywords:** AIL, Home care program, Multiple myeloma, Home therapy

## Abstract

Advanced age at diagnosis, comorbidities and invalidating symptoms can debilitate multiple myeloma (MM) and AL amyloidosis (AL) patients, potentially precluding access to optimal therapy. The *Associazione Italiana contro le Leucemie*, *Linfomi e Mieloma* (AIL) has been providing home care to hematological patients in Italy since 1993. Between 2014 and 2023, 83 MM and AL patients received home infusion therapy through AIL in Bologna. Half were newly diagnosed, and half had undergone 1–7 prior lines of therapy. The median age was 79 years and 56% of patients had a performance status ≥ 2. The most frequently administered drugs included bortezomib (82%), daratumumab (14%), and bendamustine (9%). During the median 116-day therapy duration (range 1–750), patients received a median of 12 drug administrations and 12 home visits (range 1–59). Two-thirds of the patients never attended the outpatient clinic during home therapy. Ten newly diagnosed transplant-eligible patients were able to receive standard induction, restore their performance status, and proceed to transplantation. Twenty-one newly diagnosed transplant-ineligible patients received bortezomib and dexamethasone (with or without melphalan). At relapse, they were able to receive outpatient infusion therapy in 5 out of 11 cases. Elderly, frail patients, and patients in later relapse stages could maintain their quality of life, avoiding distressing hospital visits and remaining at home with their families while still receiving active treatment. Home therapy proved feasible in the 22 patients with AL in this study as well. Despite the limitations of a single-center retrospective data collection, our results show that home care in these patients is safe and feasible. We hope these preliminary findings may encourage the implementation of such programs by health policies in the future.

**Supplementary Information:**

The online version contains supplementary material available at 10.1007/s10238-025-01683-2.

## Introduction

The *Associazione Italiana contro le Leucemie-Linfomi e Mieloma* (Italian Association against Leukemia, Lymphoma, and Myeloma—AIL) was founded in 1969 and is currently active in 83 cities across Italy. AIL was originally focused on funding scientific research and supporting the establishment of specialised hematology departments across the national territory. Over the years, AIL has expanded its mission with the aim of assisting patients with hematological conditions and their families, providing essential services tailored to their needs, with the goal of improving their quality of life. Specifically, among the proposed initiatives, a home care program has been established since 1993, now available in 30 Italian cities, with the aim of providing medical care to onco-hematology patients, reducing stress and anxiety related to the disease and logistical organization, and providing greater support to patients and their caregivers. As a spontaneous initiative of a nonprofit association, the home care program is dedicated to hematologic patients afferent to the local sanitary districts, and aims to respond primarily to the needs of patients who have difficulty reaching the hospital facility, elderly, or nonambulatory patients, and to provide assistant care, including the home administration of anticancer drugs.

Multiple myeloma (MM) is the second most common hematological malignancy, with an estimated 4.5–6.0 case per 100,000 people per year in Europe. It predominantly affects older adults, with a median age at diagnosis of 69 years, and a 2:1 incidence ratio in men compared with women. Clinically, it may manifest with bone disease, anemia, renal failure, and hypercalcemia [1]. AL amyloidosis (AL) is a rarer plasma cell disorder that can affect the heart, kidneys, peripheral nervous system, liver, and gastrointestinal tract [2].

In view of the typical characteristics of the disease, MM patients are often elderly, and carriers of multiple comorbidities. In this regard, the International Myeloma Working Group (IMWG) has developed a frailty score based on age, performance status, functional activity, and comorbidities, so as to modulate the anti-MM treatment according to the patient’s characteristics [3]. For example, in case of frail patients treatment should prioritise safety and the improvement of the quality of life; therefore, dose reductions and lower-intensity therapeutic approaches are frequent in such patients. Conversely, fit patients should receive more intensive treatments aimed at achieving deep responses and prolonged remission. However, mobility issues and complex social circumstances can prevent some patients from accessing outpatient treatments, such as daratumumab- and/or bortezomib-based therapies, not necessarily due to their frailty score, but rather due to the challenges of frequent hospital visits, that burden patients and their caregivers, and ultimately results in a reduced adherence to the clinical care pathway. In addition, organ involvement can significantly weaken both MM and AL patients, even younger ones. In particular, bone disease can severely impact mobility, making hospital visits difficult. However, advances in treatment have led to remarkable responses, enabling full recovery of physical function and extended survival [4]. The inability to receive inpatient therapy should not be a barrier to patients, or prevent them from accessing “full dose intensity” treatment, which can result in long remissions and survival benefits, as is the case for fit individuals. Moreover, advanced stages of the disease may profoundly debilitated patients. When traveling to the hospital becomes impossible, treatment options may be severely limited.

Therefore, the home care program offers several advantages to patients with MM and AL, providing them with high-quality, hospital-level treatment, and preventing them from limiting their therapies by relying exclusively on lower-intensity oral therapies, which are often less effective. In recent years, home care for hematological patients, particularly those with MM, has been implemented in various European countries [5–9]. The “hospital-at-home” model was successfully introduced during the COVID-19 pandemic to reduce infection risks in the hospital setting [10,11].

The aim of this study was to evaluate the benefits of home-administered therapy for patients with plasma cell disorders. We therefore collected data on patients enrolled in the AIL home care program for infusion therapy over the past decade. We also included patients with AL, a group that is underrepresented in the existing literature on home care.

## Methods

AIL Bologna is based at the “Seràgnoli” Institute, OU of Hematology, IRCCS Azienda Ospedaliero-Universitaria di Bologna, Policlinico Sant’Orsola (IRCCS AOUBO). Among the initiatives promoted and supported by this nonprofit association is the home care service. The AIL home care program is available for patients who have difficulty traveling frequently to the hospital for visits, blood tests, and treatments, due to impaired or reduced motility, advanced age and/or bone disease, frail patients who are unable to tolerate prolonged waiting times, or whose caregivers are unable to drive them to hospital regularly. Over time, it has also extended to patients who are discharged early from the hospital and those for whom hospitalisation is not necessary or possible.

The program is governed by an agreement between AIL Bologna, the Local Health District of Bologna (i.e., Azienda Unità Sanitaria Locale—AUSL—of Bologna) and the IRCCS AOUBO. Due to limited resources, the service is only available to patients residing in the Sanitary District of Bologna (i.e., under the jurisdiction of the AUSL of Bologna). Participation in the home care program requires the presence of a caregiver and the cooperation of the patient’s general practitioner (GP). Patients, caregivers, and GPs must sign an informed consent form before enrollment in the program. In addition, all participants signed an informed consent to participate in this data collection and expressed their consent for the conduct of the present analysis prior to their inclusion in the study. Patients eligible for the home care program are identified by hematologists working in the outpatient program of plasma cell dyscrasias and notified to AIL. Each case is discussed collegially before enrollment and during therapy within the home care program. GPs receive periodic updates on their patients and are responsible for prescribing non-oncology medications. Home care is discontinued when patients are able to safely resume outpatient care due to improved performance status thanks to treatments, or when caregivers can assist and accompany them for hospital visits. Otherwise, home care can be interrupted when infusion treatments are discontinued, either due to the completion of fixed-duration therapy or disease progression, or in case of death.

The Bologna AIL currently employs five physicians and three nurses, who perform home visits, administer therapies, blood tests, medications, and transfusions. Other procedures, such as bone marrow biopsies and diagnostic imaging tests, are performed in the hospital. A telephone line is available daily for emergencies. Non-severe complications are primarily managed at home, whenever patient safety is ensured. Severe complications or infections unresponsive to first-line antibiotics are referred to the emergency department. Oncologic drugs are prescribed by AIL hematologists and prepared in the hospital pharmacy. Funding for oncology drugs comes from the AUSL of Bologna, while other expenses are covered by AIL.

In this analysis, we retrospectively gathered data from patients with MM and AL who received home infusion therapy with AIL Bologna, starting treatment between 2014 and 2023. The data cutoff was March 15, 2024. Baseline patient characteristics were assessed at the start of home therapy, including age, performance status, functional status, and comorbidities. We assessed the Eastern Cooperative Group (ECOG) performance status [12], the Charlson’s Comorbidity Index (CCI) [12], the Katz activities of daily living (ADL) score [13], and the Lawton instrumental activities of daily living (IADL) score [14]. The IMWG frailty score was then calculated for newly diagnosed, transplant-ineligible patients. These scores are outlined in the Supplementary Tables S1-5. The Pearson R correlation index was calculated to evaluate the correlation between age and performance status or CCI.

Data on disease and therapy characteristics were collected, including disease type, organ involvement, phase and refractoriness status, type, duration, response (as per IMWG criteria) [15], and toxicity of home therapy. The number of home calls and hospital visits were assessed, along with data on transfusions and zoledronic acid therapy.

Data on patient follow-up and subsequent therapies were collected. The Kaplan–Meier survival analysis was performed to calculate progression-free survival (PFS) and overall survival (OS).

## Results

### Baseline patients’ characteristics

Infusion therapy was started in 83 patients with plasma cell disorders between 2014 and 2023. All of them provided consent to share their data for research purposes. Sixty-one patients had MM, 19 had AL, and three were diagnosed with both diseases (Table 1).

**Table 1:** Baseline patients’ characteristics at the start of home therapy

Baseline characteristics	*N* = 83
Females [*n* (%)]	44 (52)
Median age, years (range)	79 (43;91)
Age > 70 years [*n* (%)]	64 (77)
MM [*n* (%)]	61 (73)
AL [*n* (%)]	19 (23)
MM and AL [*n* (%)]	3 (4)
Newly diagnosed [*n* (%)]	41 (49)
Newly diagnosed MM, transplant-eligible [*n* (%)]	11 (13)
Newly diagnosed MM, transplant-ineligible [*n* (%)]	21 (25)
Relapsed/refractory [*n* (%)]	42 (51)
1 previous line [*n* (%)]	24 (29)
> 1 previous line [*n* (%)]	18 (22)
ECOG PS, median (range)	2 (0;4)
CCI, median (range)	4 (0;8)
Katz ADL score, median (range)	6 (0;8)
IADL score, median (range)	5 (0;8)

*ADL* activities of daily life, *AL* AL amyloidosis, *CCI* Charlson’s Comorbidity Index, *ECOG PS* Eastern Cooperative Oncology Group Performance Status, *IADL* instrumental activities of daily life, *MM* multiple myeloma, *n* number

Among these, thirty-nine patients (46%) were male, the median age was 79 years (range 43–91), and 76% of patients were older than 70 years. The median ECOG performance status was 2, and about a third of patients had an ECOG of 3 or higher. ECOG was not significantly correlated with age (*p* = 0.355). The median CCI was 4, and was weakly but significantly correlated with older age (*R* = 0.449, *p* < 0.001). The median Katz ADL score was 6, and the median Lawton IADL score was 5, reflecting a relatively independent, yet moderately low-functioning population.

Among 41 newly diagnosed patients, 9 had AL and 32 had MM. Of the newly diagnosed MM cases, 11 were eligible for transplantation. Among the 21 transplant-ineligible patients, none were fit, 3 were intermediate-fit, and 16 (86%) were frail, according to the IMWG frailty score. Additionally, 42 patients had relapsed/refractory (RR) disease, with 1 to 7 previous lines of therapy. Twenty-four patients had received a single prior line, 18 had multiple, and 33 were refractory to lenalidomide.

Clinical features, according to the CRAB (hypercalcemia, renal dysfunction, anemia, and bone disease) criteria, were as follows: bone disease was present in 50 patients (62%), anemia in 34 (44%), renal insufficiency in 18 (22%), and hypercalcemia in three (4%). Five patients (6%) had extramedullary disease.

### Home care characteristics

As per AIL organisation, and in accordance with the agreement among AIL Bologna, the AUSL of Bologna and the IRCCS AOU of Bologna, the distance between patients’ homes and the hematologic department ranged from 0.5 to 39 km. The median duration of home therapy was 119 days (range 7–749). Eight patients received a second line of home therapy, with a median duration of 81 days (range 2–641).

**Fig. 1:**
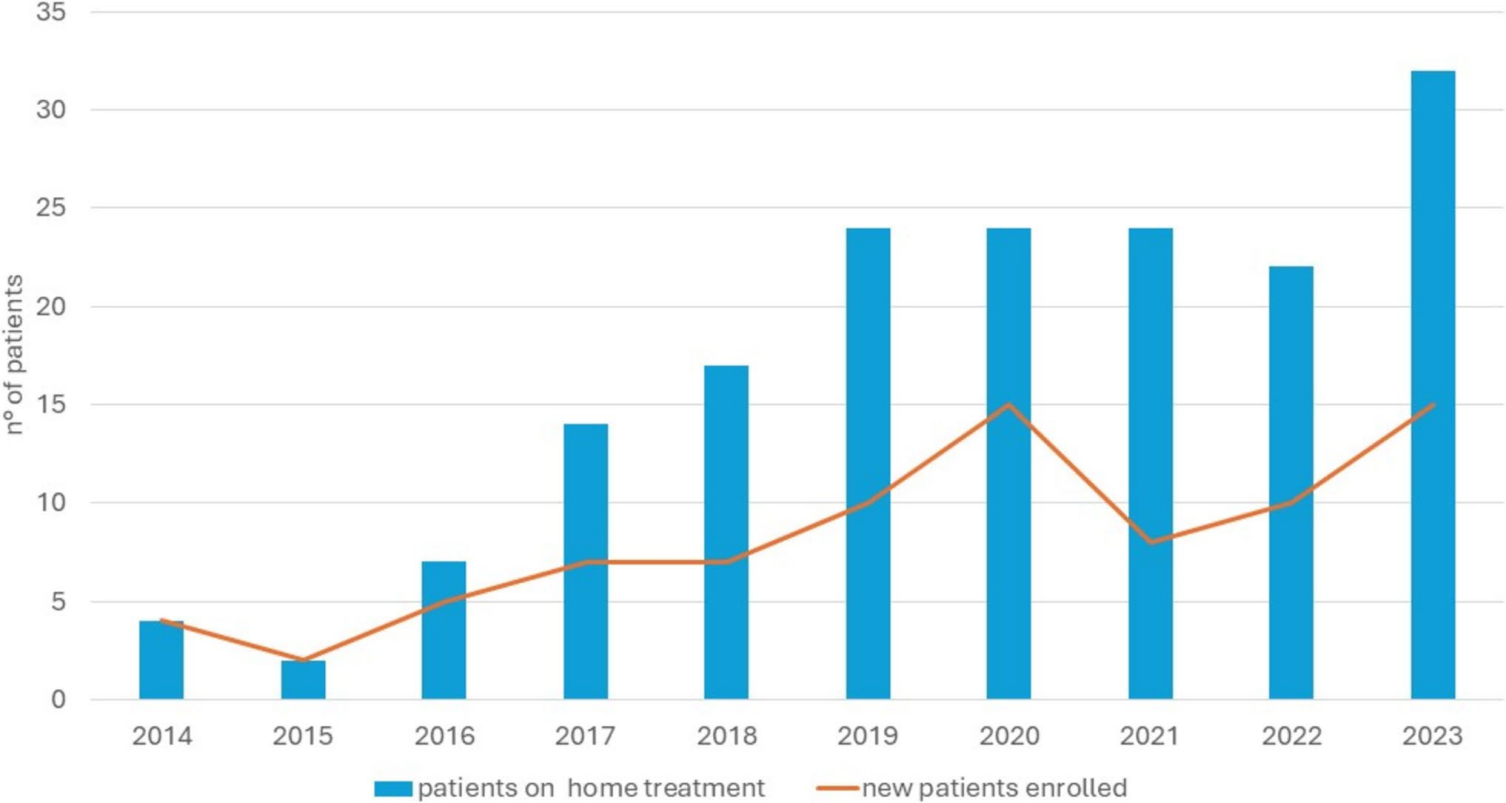
Number of new patients enrolled (orange line) and number of patients on active home treatment (blue column) each year

The number of patients enrolled each year progressively increased over time, with peaks of 15 new enrollments in 2020 (followed by a drop in 2021, possibly related to the COVID-19 pandemic) and in 2023. During 2023, 32 patients received home treatment with AIL (Fig. 1).

Each patient received a median of 12 home visits by AIL nurses and/or doctors during the whole duration of home therapy (range 1–59), and 54 of them (65%) never attended the hematology clinic. Among the remaining 29 patients, the median number of hospital visits while on active home treatment was 1.5 (range 1–10).

### Treatments

**Fig. 2:**
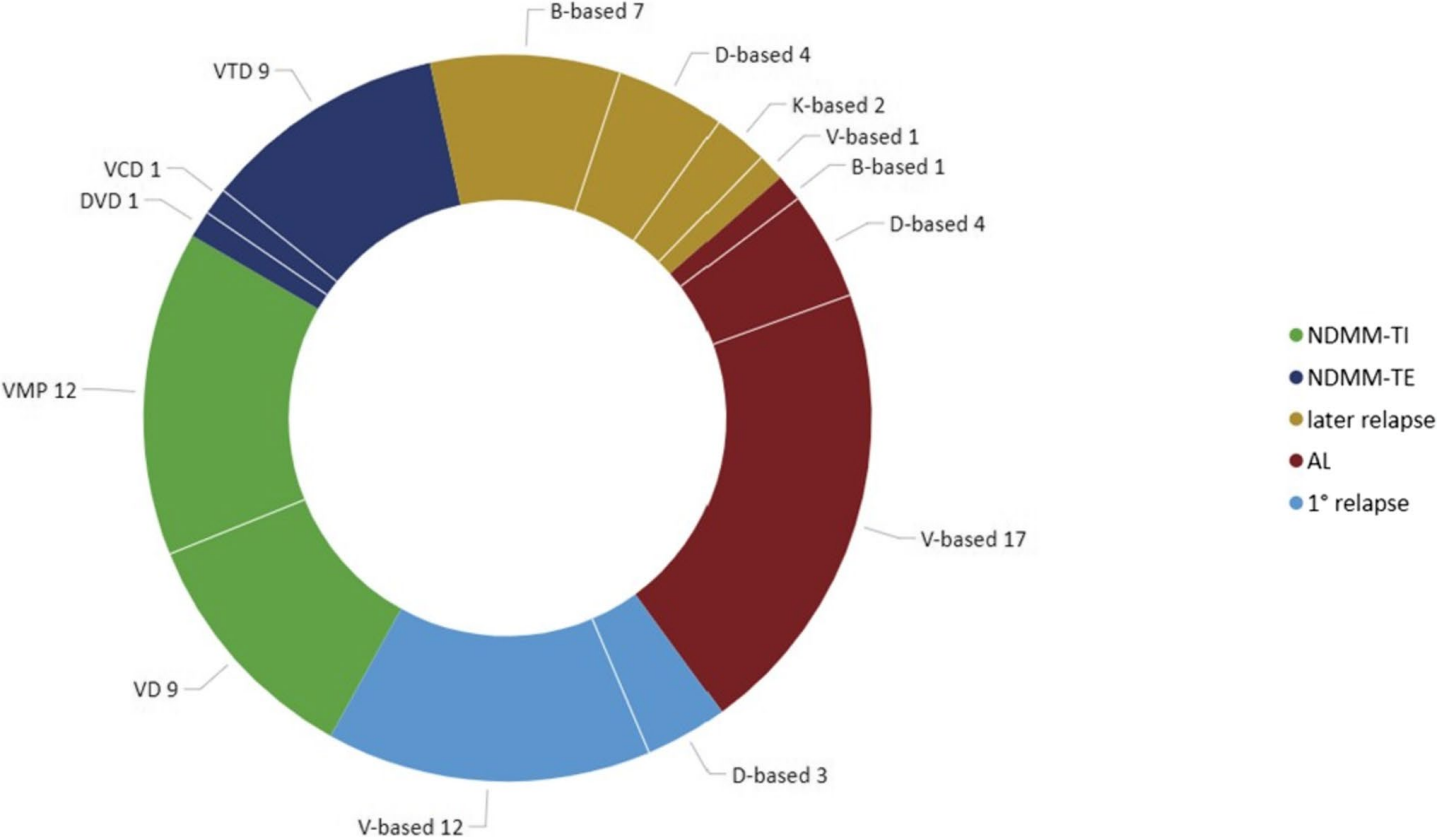
Types of therapies administered as first line of home treatment for the main categories of patients. All patient categories but those in red (labeled as AL), refer to MM patients. AL: AL amyloidosis, B: bendamustine, D: daratumumab, DVD: daratumumab-bortezomib-dexamethasone, K: carfilzomib, NDMM-TE: newly diagnosed multiple myeloma—transplant eligible, NDMM-TI: newly diagnosed Multiple Myeloma—transplant ineligible, VCD: bortezomib-cyclophosphamide-dexamethasone, VD: bortezomib-dexamethasone, VMP: bortezomib-melphalan-prednisone, VTD: bortezomib-thalidomide-dexamethasone

Home therapy was initiated a median of 5 months after diagnosis (range 0–216 months). Figure 2 depicts the therapeutic combinations of home care therapy provided by AIL. Most patients received bortezomib-based therapies (68 patients, 82%). The second most frequently administered drug was daratumumab (12 patients, 14%). Bendamustine was administered in 9 patients (11%) and carfilzomib in three patients (4%). Both bortezomib and daratumumab were administered subcutaneously, while bendamustine and carfilzomib were delivered intravenously. The most frequent combinations were bortezomib-melphalan-prednisone (23 patients, 27%), and bortezomib-dexamethasone (26 patients, 31%). Ten patients received bortezomib and dexamethasone with thalidomide (or, in one case, cyclophosphamide) as induction therapy before autologous transplantation. Therapy was prescribed at a reduced dose and/or schedule in 34 patients (40%).

Of the 8 patients who received a second line of home infusion therapy with AIL, six received daratumumab, one received carfilzomib in association with lenalidomide and dexamethasone, and one received bendamustine. The main aspects of home care are outlined in Table 2.

**Table 2:** Characteristics of home care: treatments received (number, type, and duration) and logistics

	*N* = 83
Median distance patients’ home-outpatient hematologic dpt, km (range)	7 (0.5;39)
*Lines of home infusion therapy with AIL*	
One	75 (90)
Two	8 (10)
*Drug administered (all lines)*	
Bortezomib [*n* (%)]	68 (82)
Daratumumab [*n* (%)]	18 (22)
Bendamustine [*n* (%)]	9 (11)
Carfilzomib [*n* (%)]	3 (4)
*Type of therapy (first line)*	
VD	26 (31)
VMP	23 (28)
VTD	9 (11)
Daratumumab	8 (10)
BD	4 (5)
BV	4 (5)
KD	2 (2)
PVD	2 (2)
DVD	2 (2)
DVCD	1 (1)
DRD	1 (1)
VCD	1 (1)
*Type of therapy (second line)*	*N* = 8
Daratumumab	6 (75)
KRD	1 (12)
Bendamustine	1 (12)
	*N* = 83
Home visits per patient (total), median (range)	12 (1;59)
Received transfusions [*n* (%)]	19 (23)
Received zoledronic acid [*n* (%)]	19 (23)
Never attended outpatient hematologic dpt during home therapy [*n* (%)]	54 (65)
Duration of AIL therapy, days, and median (range)	116 (7;750)
Total duration of home care, days, and median (range)	229 (7;2075)

*AIL* Associazione Italiana contro le Leucemie, Linfomi e Mieloma, *BD* bendamustine-dexamethasone, *BV* bendamustine-bortezomib, *DRD* daratumumab-lenalidomide-dexamethasone, *DVD* daratumumab-bortezomib-dexamethasone, *DVCD* daratumumab-bortezomib-cyclophosphamide-dexamethasone, *dpt* department, *KD* carfilzomib-dexamethasone, *KRD* carfilzomib-lenalidomide-dexamethasone, *n* number, *PVD* pomalidomide-bortezomib-dexamethasone, *VCD* bortezomib-cyclophosphamide-dexamethasone, *VD* bortezomib-dexamethasone, *VMP* bortezomib-melphalan-prednisone, *VTD* bortezomib-thalidomide-dexamethasone

AIL home care also provided blood testing, medications, transfusions, and supportive care. Nineteen patients received 1 to 16 zoledronic acid administrations with AIL (median: 5) and 19 received erythrocyte and/or platelet transfusions. Overall, the median duration of home care was 224 days (range 7–2337).

### Response and toxicity

Considering the first line of home-administered therapy, the overall response rate was 58% (78% in newly diagnosed patients), and a serological complete response was obtained in 15 patients (19%). Nine patients (11%) progressed during therapy without ever achieving a hematological response. The median duration of the second line of home-administered therapy was too short to evaluate the response to treatment.

Twenty-one patients (28% of evaluable patients) experienced at least one infectious complication of grade 3 or higher in 11. Nineteen patients received a median of 2 (range 1–27) erythrocyte transfusions at home with AIL. Three of them also received platelet transfusions (one, three, and 9 units, respectively).

Twenty-nine patients (37%) accessed the emergency department at least once, mostly because of infections or renal failure. Twenty-five patients (31%) were hospitalised at least once, and the median duration of hospitalisation was 12 days (range 2–149). A worsening of ECOG performance status was observed in 29 patients (35%).

A dose and/or schedule reduction of bortezomib was required in 19 patients (23%) during therapy.

Considering the second line of therapy, two patients experienced grade 3 or higher hematologic toxicity and two presented grade 3 or higher infectious complications.

### Outcomes ad follow-up

Thirty patients were alive at data cutoff (37%). Median PFS was 12 months (22 months in newly diagnosed patients) and OS 24 months. The small sample size did not allow an OS comparison between different categories of patients. Fourteen patients were still receiving home infusion therapy with AIL (nine of them were on their first line of therapy with AIL and five on the second.) The main reasons for AIL home therapy discontinuation were end of therapy (35%), death (19%), improvement of performance status (16%), and progressive disease (11%).

Thirty-five patients (44%) progressed after the first line of home infusion therapy and three out of eight (37%) after the second. Thirty-five patients underwent 1 to 5 subsequent lines of therapy: 16 received infusion therapies at the hematologic department, while 19 received only oral therapies.

Forty-two patients (50%) maintained some form of AIL home assistance after the end of infusion therapy, including blood testing, transfusions, zoledronic acid infusions, and medications. Of these, 10 received subsequent infusion therapies and 17 subsequent fully oral therapies while still receiving supportive therapy and other home services with AIL, in a mixed home- and hospital-based model of care.

### AL amyloidosis

Twenty-two patients enrolled in the home care program had AL; 3 of them also had a diagnosis of MM. They had similar median age (76 years) and the same median ECOG performance status as the global cohort. Nine (39%) had newly diagnosed disease. Four AL patients received two lines of home therapy with AIL.

Eleven patients (50%) had cardiac involvement and seven (32%) had renal involvement. When evaluable, the N-terminal pro B-type natriuretic peptide-based cardiac stage was I in two cases, II in four cases, and IIIb in two cases. The renal stage was I in four cases and III in three cases. Four patients (18%) had hepatic involvement, three (14%) had gastrointestinal involvement, and one had peripheral nervous system involvement. Five patients had single-organ involvement, while eight had multisystemic amyloidosis, involving two organs in four patients, three organs in three patients, and four organs in one patient. Data on organ involvement were not evaluable in nine patients.

Bortezomib was the most frequently administered drug (9 patients). Daratumumab was administered to four patients during their first line of home therapy with AIL and to three patients during their second line.

The rate of complete hematological responses was similar to that of the global cohort (22%), while the overall response rate was only 40%. Organ response was not evaluable by the current criteria, as N-terminal pro B-type natriuretic peptide was not routinely evaluable, and 24-h urine sampling was challenging in infirm patients. Twelve AL patients (52%) were referred to the emergency department during home therapy, accounting for almost half of the total referrals in the whole study population. Ten patients were hospitalised (43%), for a median of 20 days. The small sample size prevented a proper statistical comparison between AL and MM patients.

## Discussion and conclusion

Novel therapies for MM, including proteasome inhibitors, immunomodulatory drugs and, more recently, monoclonal and bispecific antibodies, antibody–drug conjugates, and CAR-T cells, have significantly transformed the prognosis of the disease. These treatments can elicit remarkable responses, lead to prolonged remissions, and dramatically extend survival in what was once a rapidly fatal condition [4]. The introduction of daratumumab has also redefined the treatment landscape for AL, yielding impressive rates of hematologic and organ responses [2].

However, most of these new therapies require subcutaneous or intravenous administration and are typically restricted to the hospital setting. They are generally delivered in outpatient clinics, with schedules ranging from biweekly to monthly. MM patients often face difficulties traveling to hospitals for treatment because of bone disease and/or advanced age at diagnosis. AL patients are frequently even more debilitated, particularly in cases of cardiac involvement. Also, complex social circumstances can further hinder the access to outpatient care, especially for elderly patients, whose caregivers, when available, are often similarly aged partners.

The prevalence of MM has risen globally in recent years, due to the availability of effective therapies inducing extended survival [16,17]. Consequently, outpatient clinics are frequently overcrowded, with long waiting times for patients and an increased workloads for hospital staff.

In recent years, home therapy has been implemented across countries to address these challenges. The Multiple Myeloma Clinical Unit at the “Seràgnoli” Institute of Hematology in Bologna takes care for approximately 800 patients, of whom about 450 on active infusion treatment. Over the past decade, 83 MM and AL patients have received infusion therapy at home through the AIL Bologna home care program. Fourteen of them are still under active treatment with AIL. Half of the patients in this study were newly diagnosed, the other half had relapsed or refractory disease. Notably, the therapies administered within the program reflected the advancements in MM and AL treatment over the years, and this inevitably affected the results registered in this analysis. As an example, the most commonly administered regimen since the establishment of the AIL home care program was the combination of bortezomib, melphalan, and dexamethasone, which was the standard of care for transplant-ineligible MM patients until the recent introduction of daratumumab. This patient group accounted for approximately one-third of the study population in this analysis.

The majority of patients was over 70 years old, although about one-quarter were younger. Interestingly, the ECOG performance status did not correlate strongly with age, highlighting the influence of organ involvement on functional ability. Most of the patients never visited the hematology clinic once they started home treatment. This was made possible because AIL provided at-home blood tests, medications, and ancillary treatments such as bisphosphonate infusions and transfusions, in addition to the anticancer therapy. The close collaboration between AIL and hospital staff was essential to the success of this approach. Dose and schedule modifications were carried out according to the frailty of the patients and the side effects deriving from therapy. Hematologic responses were favorable, consistent with the outcomes reported in the literature for the therapeutic combinations used in the period considered [18,19], particularly given that most patients were elderly and/or frail [3]. The safety profile was acceptable. The median PFS was one year across the entire cohort and nearly two years in newly diagnosed patients. The median OS was two years.

Follow-up data allowed us to identify different categories of patients who benefited from home therapy. The younger, fitter patients were able to receive standard-of-care induction therapy, restore their performance status, and proceed to transplantation in all but one case. These patients subsequently received consolidation and maintenance therapy as outpatients. Older, fit patients received standard first-line therapy, and at relapse, were able to receive outpatient infusion therapy in a significant proportion of cases (5 out of 11 newly diagnosed, transplant-ineligible patients who relapsed after initial home therapy). For elderly, frail patients, home care helped to maintain their quality of life by sparing them and their caregivers from distressing hospital visits. This was also true for patients in later stages of relapse, whose performance status was strongly affected by the disease, allowing them to remain at home with their families while still receiving active treatment. Moreover, the benefit of receiving care and treatment at home may also potentially translate into a reduction in the risk of contracting nosocomial infections in cytopenic patients.

Notably, we also included AL patients in the analysis, as these are underrepresented in existing published reports on home care. Interestingly, our data indicated that home therapy is feasible for these patients, who are often debilitated by cardiac, gastrointestinal, and/or autonomic involvement, but can achieve favorable hematologic and organ responses with appropriate treatment.

Noteworthy, though a head-to-head comparison among patients treated in the hospital setting versus those treated at home was not feasible for this analysis, the home care program proved to be feasible, resulting in a reduction of patients visiting hospitals for blood draws, therapy administration, or in many cases, medical consultations. Additionally, the reduction in hospital visits also relieved caregivers from the need to transport patients to the clinic. Furthermore, patients benefited from a lower staff-to-patient ratio in case of urgent needs. This significantly impacted patients and caregivers’ satisfaction. Indeed, the home care program provided by AIL also includes a satisfaction survey of patients and their families (data not shown). According to the statistics published annually by AIL, the service has resulted in a reduction in illness-related stress, increased compliance and continuity of care, avoiding exposure to hospital environments, and ensuring a certain degree of autonomy for the caregivers. In addition, families felt more easily involved, which allowed for better management of the disease and its emotional impact, benefiting both the patient and their caregivers. Also, though a pharmacoeconomic analysis was not performed in our study, the one conducted by Touati and colleagues demonstrated significant cost savings in favor of home care.^9^

Certainly, our study has some limitations. First, due to limited resources, AIL home care is available only to patients residing within the Bologna Sanitary District, although the Institute of Hematology “Seràgnoli” serves as a hub for a relatively large region, as well as an attractive hub for patients from outside the region. In view of this, many eligible patients could not be enrolled in the program. Second, the retrospective, single-center design of this study and the small sample size limit the generalisability of the findings. The small cohort also precluded a statistical comparison of safety and efficacy between MM and AL patients receiving home-administered therapy. Nevertheless, we demonstrated that home-based infusion therapy is both safe and effective for MM and AL patients in our center.

Importantly, data regarding patients treated in the first years since the establishment of the home care program do not fully reflect the current standards of care for MM and AL. However, 18 patients received daratumumab at home without significant complications, suggesting that newer monoclonal antibody therapies can also be safely administered in this setting, opening a window into the possibility and feasibility of home care programs in the current and future therapeutic scenario.

Moreover, this study focused on infusion therapy, which is among the most resource-intensive treatments for both patients and healthcare systems. Nevertheless, home care is also valuable for patients receiving oral therapies. To optimise resource allocation, we typically reserve AIL home care for patients requiring infusion therapy, taking advantage of the multidisciplinary approach and the presence of hematologists and nurses specialised in hematologic diseases, while patients on oral treatments may be referred to other home care services, either through their general practitioners or non-hematologic charitable organisations.

Collectively, our data show that home care is safe and feasible. In view of this, we advocate for a broader implementation of home care programs for hematologic patients, especially those with chronic and debilitating diseases like MM and AL, as part of a strategy for better resource management and personalised care in this rapidly evolving field. We hope that the preliminary findings from this retrospective pilot study will encourage such implementation. Additionally, we have recently launched a single-center prospective study for hematological patients receiving home care upon early discharge from hospital, whose results will hopefully provide further evidence on the advantages of home care in these patients. Ideally, these and further results may serve as a basis for the implementation of community-based health services in the future. A revision of current health policies may help reduce geographic and socioeconomic barriers, progressively expanding access to larger numbers of patients, with the ultimate goal of reducing the impact of hematologic diseases on patients and their families.

## Supplementary Information

Below is the link to the electronic supplementary material.

Supplementary file1 (DOCX 22 kb)

## Data Availability

The data supporting the findings of this study are available via Zenodo platform (DOI: 10. 5281/zenodo.14038968).
