# Supplementary material for: Ten-year experience on home care for patients with plasma cell disorders: bringing optimal therapy home
Source: Clin Exp Med. 2025 May 27;25(1):179. doi: 10.1007/s10238-025-01683-2 (PMC12116699; doi:10.1007/s10238-025-01683-2)
Supplement: Supplementary file 1 — Supplementary file1 (DOCX 22 kb) [file 10238_2025_1683_MOESM1_ESM.docx]

SUPPLEMENTARY MATERIALS

| Score | Description |
| --- | --- |
| 0 | Fully active |
| 1 | Restricted in physically strenuous activity but ambulatory and able to carry out work of a light or sedentary nature |
| 2 | Ambulatory and capable of all self-care but unable to carry out any work activities. Up and about ≥ 50% of waking hours |
| 3 | Capable of only limited self-care, confined to bed or chair ≥ 50% of waking hours |
| 4 | Completely disabled. Cannot carry on any self-care. Totally confined to bed or chair |
| 5 | Death |

Table S1. Eastern Cooperative Oncology Group (ECOG) Performance Status. The ECOG score can estimate the ability of a patient to tolerate anti-cancer therapy. Adapted from Oken MM, Creech RH, Tormey DC, et al. Toxicity and response criteria of the Eastern Cooperative Oncology Group. Am J Clin Oncol. 1982;5(6):649-655.

| **Variable** | **Score** |
| --- | --- |
| Age 50-59 years | 1 |
| Age 60-60 years | 2 |
| Age 70-79 years | 3 |
| Age ≥ 80 years | 4 |
| Myocardial infarction | 1 |
| Chronic heart failure | 1 |
| Peripheral vascular disease | 1 |
| Cerebrovascular accident or transient ischemic attack | 1 |
| Dementia | 1 |
| Chronic Obstructive Lung Disease | 1 |
| Connective tissue disease | 1 |
| Peptic ulcer disease | 1 |
| Mild liver disease | 1 |
| Moderate or severe liver disease | 3 |
| Uncomplicated diabetes mellitus requiring treatment | 1 |
| Diabetes mellitus complicated by end-organ damage | 2 |
| Hemiplegia | 2 |
| Moderate to severe chronic kidney disease | 2 |
| Localised solid tumour | 2 |
| Metastatic solid tumour | 6 |
| Leukaemia | 2 |
| Lymphoma | 2 |
| Acquired ImmunoDeficiency Syndrome | 6 |

Table S2. Charlson’s Comorbidity Index (CCI). Each variable is assigned a score from 1 to 6. The total score estimates the number and severity of comorbidities.

| **Category** | **Description** | **Score** |
| --- | --- | --- |
| Eating | Gets food from plate into mouth without help. Food may be prepared by another person. Eats without assistance | 1 |
|  | Needs partial or complete assistance in eating or is fed intravenously | 0 |
| Dressing | Gets clothes from closets and drawers and puts on clothes and outer garments complete with fasteners. May have help tying shoes. | 1 |
|  | Needs help with dressing or needs to be completely dressed | 0 |
| Bathing | Bathes self completely or needs help in bathing only a single part of the body such as the back, genital area, or disabled extremity | 1 |
|  | Needs assistance in bathing more than one part of the body, getting in and out of the tub or shower, or needs total assistance | 0 |
| Transferring | Moves in and out of bed or chair unassisted (mechanical transfer aids are acceptable) | 1 |
|  | Needs help in moving from bed to chair or requires a complete transfer | 0 |
| Toileting | Goes to toilet, gets on and off, arranges clothes, and cleans genital area without help | 1 |
|  | Needs help transferring to the toilet, cleaning self or uses bedpan or commode | 0 |
| Continence | Controls bladder and bowel completely (without occasional accidents) | 1 |
|  | Is partially or totally incontinent of bowel or bladder | 0 |

Table S3. Katz’ Activities of Daily Living (ADL) scale. A score of 0 or 1 is assigned for each category. A total score of 6 indicates the patient is independent, 4 indicates the patient has moderate impairment, and 0 indicates the patient is very dependent. Adapted from Katz S. Assessing self-maintenance: Activities of daily living, mobility, and instrumental activities of daily living. Journal of the American Geriatrics Society. 1983;31(12):721-727. doi:10.1111/j.1532-5415.1983.tb03391.x

| **Category** | **Description** | **Score** |
| --- | --- | --- |
| Telephone | Operates telephone on own initiative-looks up and dials numbers, etc. | 1 |
|  | Dials a few well-known numbers | 1 |
|  | Answers telephone but does not dial | 1 |
|  | Does not use telephone at all | 0 |
| Shopping | Takes care of all shopping needs independently | 1 |
|  | Shops independently for small purchases | 0 |
|  | Needs to be accompanied on any shopping trip | 0 |
|  | Completely unable to shop | 0 |
| Food preparation | Plans, prepares and serves adequate meals independently | 1 |
|  | Prepares adequate meals if supplied with ingredients | 0 |
|  | Heats, serves and prepares meals, or prepares meals, or prepares meals but does not maintain adequate diet | 0 |
|  | Needs to have meals prepared and served | 0 |
| Housekeeping | Maintains house alone or with occasional assistance | 1 |
|  | Performs light daily tasks such as dish washing, bed making | 1 |
|  | Performs light daily tasks but cannot maintain acceptable level of cleanliness | 1 |
|  | Needs help with all home maintenance tasks | 1 |
|  | Does not participate in any housekeeping tasks | 0 |
| Laundry | Does personal laundry completely | 1 |
|  | Launders small items-rinses stockings, etc. | 1 |
|  | All laundry must be done by others | 0 |
| Transportation | Travels independently on public transportation or drives own car | 1 |
|  | Arranges own travel via taxi, but does not otherwise use public transportation | 1 |
|  | Travels on public transportation when accompanied by another | 1 |
|  | Travel limited to taxi or automobile with assistance of another | 0 |
|  | Does not travel at all | 0 |
| Medications | Is responsible for taking medication in correct dosages at correct time | 1 |
|  | Takes responsibility if medication is prepared in advance in separate dosage | 0 |
|  | Is not capable of dispensing own medication | 0 |
| Finances | Manages financial matters independently, collects and keeps track of income | 1 |
|  | Manages day-to-day purchases, but needs help with banking, major purchases, etc. | 1 |
|  | Incapable of handling money | 0 |

Table S4. Lawton-Brody Instrumental Activities of Daily Living (IADL) scale. A score of 0 or 1 is assigned for each category. The total score ranges from 0 (low function, dependent) to 8 (high function, independent). Adapted from Lawton MP, Brody EM. Assessment of older people: Self-maintaining and instrumental activities of daily living. *Gerontologist*. 1969;9(3):179-186. doi:10.1093/geront/9.3_Part_1.179

| **Variable** | **Value** | **Score** |
| --- | --- | --- |
| Age | ≤ 75 years | 0 |
|  | 76-80 years | 1 |
|  | > 80 years | 2 |
| ADL | > 4 | 0 |
|  | ≤ 4 | 1 |
| IADL | > 5 | 0 |
|  | ≤ 5 | 1 |
| CCI | ≤ 1 | 0 |
|  | > 1 | 1 |

Table S5. International Myeloma Working Group Frailty Score. A score is assigned for each variable. A total score of 0 identifies a fit patient, a score of 1 an intermediate-fit patient, and a score of 2 or higher a frail patient. ADL: Katz’ Activities of Daily Living; CCI: Charlson’s Comorbidity Index; IADL: Lawton Instrumental Activities of Daily Living. Adapted from Palumbo A, Bringhen S, Mateos MV, et al. Geriatric assessment predicts survival and toxicities in elderly myeloma patients: An International Myeloma Working Group report. Blood. 2015;125(13):2068-2074. doi:10.1182/blood-2014-12-615187
